# Simultaneously Quantitative Analysis of Naringin and Its Major Human Gut Microbial Metabolites Naringenin and 3-(4′-Hydroxyphenyl) Propanoic Acid via Stable Isotope Deuterium-Labeling Coupled with RRLC-MS/MS Method

**DOI:** 10.3390/molecules24234287

**Published:** 2019-11-25

**Authors:** Taobin Chen, Hao Wu, Yan He, Wenjun Pan, Zenghao Yan, Yan Liao, Wei Peng, Li Gan, Yaohui Zhang, Weiwei Su, Hongliang Yao

**Affiliations:** 1 Guangdong Key Laboratory of Plant Resources, School of Life Sciences, Sun Yat-sen University, Guangzhou 510275, China; sysulssctb@126.com (T.C.); wuhao8@mail.sysu.edu.cn (H.W.); heyan53@mail2.sysu.edu.cn (Y.H.); panwj23@mail2.sysu.edu.cn (W.P.); yanzengh@mail2.sysu.edu.cn (Z.Y.); liaoyansysu@126.com (Y.L.); pweiyu929@126.com (W.P.); 2 Shenzhen Research Institute of Sun Yat-sen University, Shenzhen 518057, China; 3 Artis Biotech Co. Ltd., Shaoxing 312000, China; lgan@artis-standards.com (L.G.); fusudu@163.com (Y.Z.); 4 Guangdong Key Laboratory of Animal Conservation and Resource Utilization, Guangdong Public Laboratory of Wild Animal Conservation and Utilization, Drug Synthesis and Evaluation Center, Guangdong Institute of Applied Biological Resources, Guangzhou 510260, China

**Keywords:** naringin, gut microorganism, microbial metabolites, stable isotope deuterium-labeling, RRLC-MS/MS

## Abstract

Widespread in citrus fruits, naringin, a natural 2,3-dihydroflavonoid, is of particular interest to scientists and has a broad range of beneficial bioactivities to health. Orally administered naringin remains in the gut tract for a relatively long time because of its low bioavailability. Under the metabolism mediated by human gut microbiota, naringin could be an active precursor for derived metabolites to play important physiological roles. However, naringin and its metabolites are hard to accurately quantify due to severe endogenic interference. In this study, an analytical rapid resolution liquid chromatography tandem mass spectrometry (RRLC-MS/MS) method coupled with stable isotope deuterium-labeling is developed and validated to simultaneously quantify naringin as well as its major human gut microbial metabolites naringenin and 3-(4′-hydroxyphenyl) propanoic acid. By eliminating the matrix interferences, this strategy not only confirms naringenin and 3-(4′-hydroxyphenyl) propanoic acid as the predominant metabolites which contribute to the pharmacological effects of naringin but also provides a suitable choice for other flavonoid pharmacokinetics study.

## 1. Introduction

Fruits not only enrich our life with good taste but also contain abundant bioactive constituents to protect human health, since consumption of fruits can help reduce risks of various diseases [1]. Citrus fruits are easily available and popular in daily diets. Among the bioactive contents in citrus fruits, flavonoids are of particular interest to researchers due to their protective effects against inflammation, cardiovascular diseases, and cancers [2,3].

Naringin, a natural 2,3-dihydroflavonoid, presents dominantly in citrus fruits and has been reported to possess a broad range of bioactive effects on health such as protection against oxidation [4,5], hyperlipidemia [6], and neurological disorders [7,8]. In addition, our previous investigations have proven that naringin has extensive beneficial effects against respiratory diseases including inflammation [9,10], coughs [11], phlegm [12], and pulmonary fibrosis [13].

With multiple varieties and potent functions, there are about 10^14^ microorganisms, tenfold more than human cells, residing in the gut tract and playing important roles in metabolic, nutritional, physiological, and immunological activities of both health and disease conditions [14]. For most bioactive molecules in food or oral medication, human gut microbiota secret numerous enzymes to get involved in the chemical transformation processes and have certain effects on not only their pharmacokinetic behaviors but also pharmacological characteristics [15]. The results of the metabolism mediated by human intestinal microbes could be positive or negative, which is correlated to bioactivities of the subsequent metabolites [16].

With its low bioavailability, naringin is poorly absorbed into the blood circulation system [17], suggesting that orally administered naringin remains in the gastrointestinal tract for a correspondingly extended time and is inevitably influenced by intestinal microbiota. In our preliminary research, we had systematically profiled the metabolites of naringin mediated by human intestinal microbiota with an ingenious strategy that combined stable isotope-labeling and ultra-fast liquid chromatography-quadruple-time-of-flight tandem mass spectrometry (UFLC-Q-TOF-MS/MS) method [18]. Among the human gut microbial metabolites, naringenin and 3-(4′-hydroxyphenyl) propanoic acid (HPPA) have been considered the major microbial biotransformation products of naringin. Of note is that both naringenin and HPPA have been reported to possess beneficial bioactivities, among which naringenin has been seen to be effective with regards to anticancer [19], anti-inflammation [20], and neuroprotection [21], while HPPA can efficiently suppress influenza infection [22]. Therefore, it seems as though naringin could be an effective precursor in vivo to play bioactive roles via its derived metabolites from the biotransformation mediated by human gut microbiota. Thus, it is of great value to quantitatively investigate the dynamic biotransformation of naringin and its human gut microbial metabolites including naringenin and HPPA. However, it is difficult to achieve an accurate quantification of naringin and its metabolites due to strong endogenic interference.

In this study, we develop a precise and sensitive rapid resolution liquid chromatography tandem mass spectrometry (RRLC-MS/MS) method coupled with application of stable isotope deuterium-labeling to simultaneously quantify naringin, naringenin, and HPPA under the metabolism mediated by human intestinal microbes.

## 2. Results and Discussion

### 2.1. Synthesis of [2′,3′,5′,6′-D_4_]naringenin (D_4_-NE) and 3-(4′-Hydroxyphenyl)-[2′,3′,5′,6′-D_4_]propanoic Acid (D_4_-HPPA)

**Figure 1:**
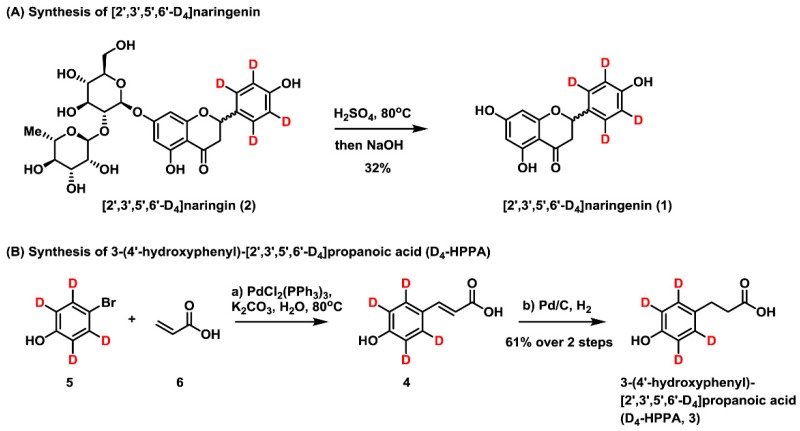
Synthesis scheme of [2′,3′,5′,6′-D_4_]naringenin (**A**) and 3-(4′-hydroxyphenyl)-[2′,3′,5′,6′-D_4_]propanoic acid (**B**).

**Figure 2:**
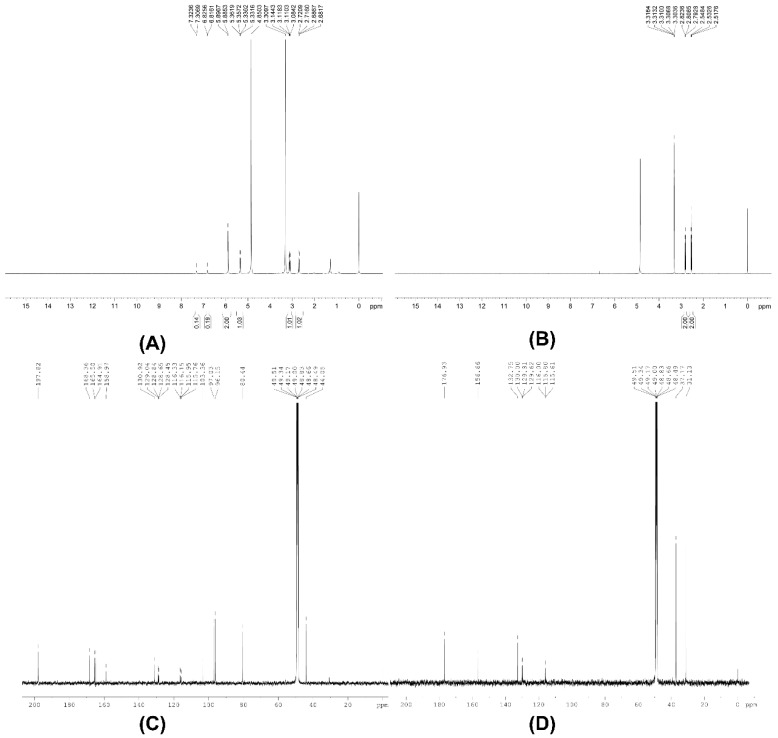
NMR spectra of [2′,3′,5′,6′-D_4_]naringenin (**A**) Proton NMR, (**C**) Carbon NMR and 3-(4′-hydroxyphenyl)-[2′,3′,5′,6′-D_4_]propanoic acid (**B**) Proton NMR, (**D**) Carbon NMR).

The synthesis scheme and ^1^H, ^13^C nuclear magnetic resonance (NMR) spectra of [2′,3′,5′,6′-D_4_]naringenin and 3-(4′-hydroxyphenyl)-[2′,3′,5′,6′-D_4_]propanoic acid are shown in Figure 1 and Figure 2, respectively. For [2′,3′,5′,6′-D_4_]naringenin, ^1^H NMR (500 MHz, MeOD) δ: 7.31 (doublet (d), *J* = 8.4 Hz, OH), 6.82 (d, *J* = 4.8 Hz, OH), 5.89 (d, *J* = 5.8 Hz, 2H), 5.35 (dd, *J* = 13.0, 2.4 Hz, 1H), 3.11 (dd, *J* = 17.0, 13.0 Hz, 1H), 2.70 (dd, *J* = 17.0, 2.4 Hz, 1H) and ^13^C NMR (125 MHz, MeOD) δ: 197.8, 168.3, 165.5, 164.9, 158.9, 130.9, and 128.7 (2C, 129.0, 128.8, 128.6, and 128.5), 116.0 (2C, 116.3, 116.1, 115.9, and 115.7), 103.4, 97.0, 96.1, 80.4, and 44.0. In addition, the melting point (m.p.) of [2′,3′,5′,6′-D_4_]naringenin was 252 °C. IR ν_max_ (cm^−1^): 3281, 3124, 1633, 1606, 1498, 1461, 1438, 1347, 1310, 1224, 1180, 1157, 1081, 1063, 963, 827, and 728. High resolution mass spectrum (HRMS, electrospray ionization source (ESI)^−^) *m/z* calculated for C_15_H_7_D_4_O_5_ [M–H]^−^ 275.0858, found 275.0846.

For 3-(4′-hydroxyphenyl)-[2′,3′,5′,6′-D_4_]propanoic acid, ^1^H NMR (500 MHz, MeOD) δ: 2.81 (triplet (t), *J* = 7.8 Hz, 2H), 2.53 (t, *J* = 7.8 Hz, 2H) and ^13^C NMR (125 MHz, MeOD) δ: 176.9, 156.6, 132.7, 129.8 (2C), 115.8 (2C), 37.1, and 31.1. The melting point was 130 °C. IR ν_max_ (cm^−1^): 3397, 3025, 2956, 2935, 2871, 2630, 1704, 1576, 1432, 1405, 1352, 1306, 1211, 1120, and 915. HRMS (ESI^−^) *m/z* calculated for C_9_H_5_D_4_O_3_ [M–H]^−^ 169.0808, found 169.0813.

In summary, the above results identified the synthetic products as [2′,3′,5′,6′-D_4_]naringenin and 3-(4′-hydroxyphenyl)-[2′,3′,5′,6′-D_4_]propanoic acid, indicating that our synthetic strategy was appropriate with expected future application in other flavonoids and phenolic acids.

### 2.2. Method Development and Optimization

For analyzing flavonoid and phenolic compounds from human biological samples, especially in fecal microbiota solution, there is a common challenge that those specimens have shown severe matrix effects from co-eluted peaks [23,24]. Thus, it is urgent to seek for a suitable strategy with excellent specificity and sensitivity to distinguish target molecules from matrix disturbances. Coupled with LC-MS, there is a stable isotope-labeling technique which has been proven to be a valid alternative for qualitative and quantitative analysis of diverse compounds with its remarkable advantages including safety, sensitivity, and versatility [25,26,27,28,29]. Referring to our previous study [18], we synthesized with the same stable isotope-labeling pattern to replace hydrogen with deuterium in the 2′, 3′, 5′, 6′ positions on the B ring of naringenin and HPPA.

As is known to us, multiple reaction monitoring (MRM) detection mode with characteristic precursors and product ions is generally considered as the gold standard for the quantification of molecules from complex biological samples [30]. Combined with the integrated advantages of high resolution, rapid speed, and excellent sensitivity, RRLC coupled with triple quadrupole mass spectrometry (TQ-MS/MS) is a better choice for quantitative analysis of compounds [31]. Hence, we developed an analytical strategy coupled with RRLC and a stable isotope deuterium-labeling method to simultaneously quantify naringin and its major metabolites naringenin and HPPA mediated by human gut microbes.

**Figure 3:**
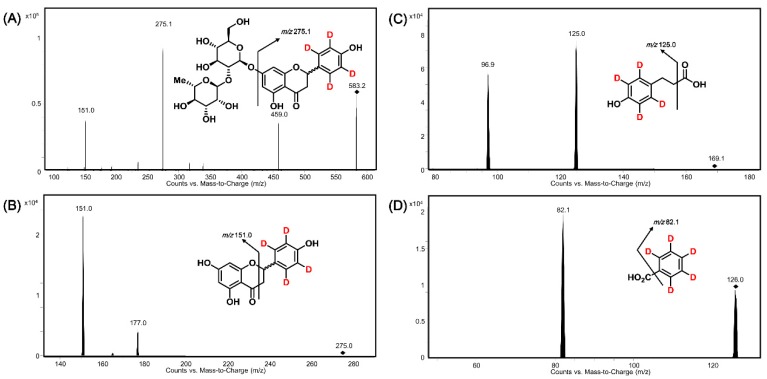
Product ion spectra and MS/MS fragmentation patterns of D_4_-NG (**A**), D_4_-NE (**B**), D_4_-HPPA (**C**) and D_5_-BA (**D**).

Sample preparation and RRLC conditions including anaerobic incubation, extraction method, mobile phase system, and so on, were employed according to our previous report with feasible adjustments [18]. For the sake of higher ion responses, we optimized the MS/MS parameters of [2′,3′,5′,6′-D_4_]naringin (D_4_-NG), [2′,3′,5′,6′-D_4_]naringenin (D_4_-NE), 3-(4′-hydroxyphenyl)-[2′,3′,5′,6′-D_4_]propanoic acid (D_4_-HPPA), internal standard (IS) benzoic-[2,3,4,5,6-D_5_] acid (D_5_-BA) (see Table 1) and MS/MS conditions such as gas temperature, gas flow, nebulizer, and capillary through injecting individual standard solutions of target compounds at a concentration of 10 μg mL^−1^. The product ion spectra and MS/MS fragmentation patterns of analytes are shown in Figure 3 and indicate that the optimized quantitative MRM transitions were 583.2→275.1, 275.0→151.0, 169.1→125.0, and 126.0→82.1 for D_4_-NG, D_4_-NE, D_4_-HPPA, and D_5_-BA, respectively.

### 2.3. Method Validation

#### 2.3.1. Specificity

**Figure 4:**
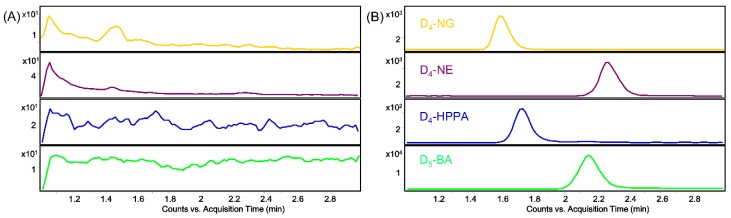
Typical MRM chromatograms of D_4_-NG, D_4_-NE, D_4_-HPPA, and D_5_-BA in blank gut microbiota solution (**A**) and blank gut microbiota solution spiked with analytes at a lower limit of quantification (LLOQ) concentration and IS (**B**).

As seen in Figure 4, typical MRM chromatograms from blank gut microbiota solution spiked with analytes at a lower limit of quantification (LLOQ) concentration and IS show sharp and satisfactory peaks with retention times (RT) at 1.6 min, 2.3 min, 1.7 min, and 2.2 min for D_4_-NG, D_4_-NE, D_4_-HPPA, and IS D_5_-BA, respectively. At the same time, there are no obvious interferential MRM signals at the RT of all the analytical targets and IS compared with the blank gut microbiota solution. This result indicates that our developed method is specific for analyzing D_4_-NG, D_4_-NE, and D_4_-HPPA simultaneously.

#### 2.3.2. Linearity

Calibrated curves with favorable regression coefficients (r > 0.99) ranged from 10 to 2000, 5 to 1000, and 2.5 to 500 ng mL^−1^ for D_4_-NG, D_4_-NE, and D_4_-HPPA, respectively, settling at 200-fold to adequately cover diverse concentrations of target analytes in co-incubating samples from different individuals. The accuracy of back-calculated concentrations from nominal values were 87.31–111.18%, 96.81–104.22%, and 89.61–110.26% for the three calibration curves of D_4_-NG, D_4_-NE, and D_4_-HPPA, respectively, indicating an accepted linearity of the established method. In addition, the limits of detection (LOD) of D_4_-NG, D_4_-NE, and D_4_-HPPA were 100 pg mL^−1^, 50 pg mL^−1^, and 25 pg mL^−1^ with signal to noise (S/N) ratios of 32.74, 41.29, and 28.63, respectively.

#### 2.3.3. Precision and Accuracy

The results of precision and accuracy validation are summarized in Table 2. The relative standard deviations (RSDs) of intra-day were 0.91–3.58%, 1.25–3.49%, and 1.45–9.90% for D_4_-NG, D_4_-NE, and D_4_-HPPA, respectively, while the relative error (RE) of intra-day varied from −9.42 to 8.73 for the three target compounds. For inter-day validation, the RSD were lower than 12% and the RE range was −8.90–9.81% for all the molecules. In summary, the above results suggest suitable precision and accuracy of our method for quantification of D_4_-NG, D_4_-NE, and D_4_-HPPA at the same time.

#### 2.3.4. Extract Recovery

The mean extraction efficiencies of D_4_-NG, D_4_-NE, and D_4_-HPPA at various concentrations were found to be 56.22–58.02%, 53.97–58.50%, and 49.53–70.69%, respectively (see Table 2). As a result, ethyl acetate was considered to be acceptable as a suitable and effective reagent for simultaneously extracting D_4_-NG, D_4_-NE, and D_4_-HPPA in human gut microbiota solutions because of its moderate polarity and solubility.

#### 2.3.5. Matrix Effect

As shown in Table 3, the matrix effects for D_4_-NG, D_4_-NE, and D_4_-HPPA were between 94.73–101.1% and 98.12–107.1% at quality control (QC) low and high concentrations, respectively. In addition, the RSDs of the three targets at the tested concentrations were no more than 3%, indicating that matrix effects were negligible for analysis with the application of stable isotope-labeling.

#### 2.3.6. Stability

Mean evaluations of D_4_-NG, D_4_-NE, and D_4_-HPPA at QC low and high concentrations under various conditions including freeze and thaw (three cycles from −80 °C to 25 °C), short-term (24 h at 25 °C) and long-term (three months at −80 °C) ranged from 94.84% to 107.3% (Table 4), indicating that there was no obvious degradation of the three target compounds. Thus, the stability of this established method was found to be satisfactory.

### 2.4. Simultaneously Quantitative Analysis of D_4_-NG, D_4_-NE, and D_4_-HPPA after Anaerobic Co-Incubation of D_4_-NG with Human Gut Microbiota Solution

The established stable isotope deuterium-labeling coupled with RRLC-MS/MS strategy was well applied to quantitative analysis after incubation of naringin with human fecal microbe solutions. As we determined, with the help of deuterium-labeling, there was no baseline interference for analysis [32,33], demonstrating that stable isotope-labeling was effective to eliminate matrix interferences from biological samples and be capable of analyzing flavonoid and phenolic compounds precisely and sensitively.

**Figure 5:**
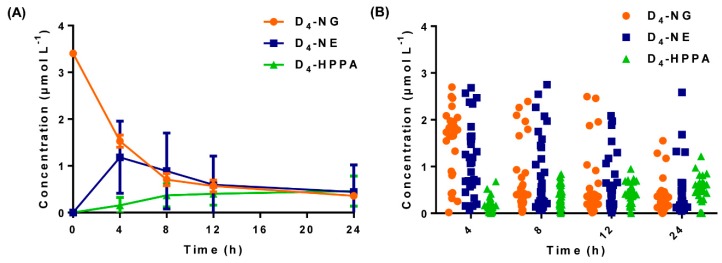
Concentration–time curves ((**A**), mean ± SD, *n* = 30) and scatter plots ((**B**), *n* = 30) of D_4_-NG, D_4_-NE, and D_4_-HPPA after anaerobic co-incubation of D_4_-NG with human gut microbiota solution.

**Figure 6:**
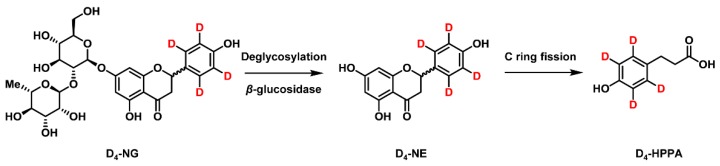
Proposed metabolic pattern of naringin mediated by human gut microbiota.

In order to systematically profile the dynamic biotransformation courses of naringin and its microbial metabolites, we detected their concentrations four sequential times within 24 h. As shown in Figure 5a, D_4_-NG underwent ongoing degradation while D_4_-HPPA continuously increased over time after incubation. Of note is that D_4_-NG was consumed after 4 h, indicating a proposed metabolic pattern of naringin mediated by human gut microbiota (Figure 6). In general, naringin was first hydrolyzed to its aglycone naringenin under the *β*-glucosidases secreted by microorganisms [34,35] and then underwent C ring fission to generate 3-(4′-hydroxyphenyl)propanoic acid [36,37]. However, the enzyme profiling which catalyzes C ring fission is still unclear and deserves in-depth research.

After 24 h, the total concentration of D_4_-NG (0.3579 μmol L^−1^), D_4_-NE (0.4418 μmol L^−1^), and D_4_-HPPA (0.4605 μmol L^−1^) was 1.2602 μmol L^−1^, which was about a 40% ratio compared to the administrated dose of D_4_-NG. Hence, naringenin and HPPA could be the major metabolites of naringin under the metabolism of human intestinal microbiota, which is in accordance with our best knowledge that gut microorganisms mainly proceed reduction and hydrolysis to produce low molecular weight and polarity compounds [15,38]. Considering the bioactivities of naringenin [19,20,21] and HPPA [22], gut microbiota could in part play an important role in regulating the pharmacological effects of naringin through biotransformation in vivo.

Of note is that the metabolic profile was significantly discrete among various individuals (Figure 5B). Due to the complexity of intestinal microecology, different compositions of bacteria show diverse physiological activities [39,40]. For decades, although the gut bacterial metabolism of naringin has been reported [41,42,43,44,45,46], the metabolic association between naringin and microbial groups is yet to be uncovered and is deserving of further research through multiple omics.

## 3. Experimental Procedures

### 3.1. Chemicals and Reagents

Potassium carbonate, sodium sulfate, sodium hydroxide, methanol, and sulfuric acid were purchased from the Guangzhou Chemical Reagent Factory (Guangzhou, China). Acrylic acid, bis(triphenylphosphine)palladium(II) chloride, palladium on carbon (30 *wt*%), and LC-MS grade formic acid were obtained from Sigma-Aldrich Co. (St. Louis, MO, USA). [2′,3′,5′,6′-D_4_] bromophenol was provided by Artis Biotech Co. Ltd. (Shaoxing, China). LC-MS grade methanol was acquired from Fisher Scientific Inc. (Fair Lawn, NJ, USA). Ethyl acetate was produced by Honeywell B&J (Morristown, NJ, USA). General anaerobic medium (GAM) broth was supplied by Nissui Pharmaceutical Inc. (Tokyo, Japan). Benzoic-[2,3,4,5,6-D_5_] acid was provided by Toronto Research Chemicals (Toronto, ON, Canada). Deionized water was prepared using the Milli-Q system (Millipore Corporation; Billerica, MA, USA).

### 3.2. NMR, Infra-Red Spectra and Melting Point Analysis

^1^H (500 MHz) and ^13^C (125 MHz) NMR spectra were acquired on a Bruker AV-500 spectrometer (Bruker, Billerica, MA, USA). Chemical shifts were reported in parts per million (ppm) with values relatively compared with the internal methanol-*D* (3.31 and 49.00 ppm for ^1^H and ^13^C, respectively). Abbreviations of signal coupling are s, d, t, q, and m for singlet, doublet, triplet, quartet, and multiplet, respectively. IR spectra were recorded using an FT-IR spectrometer (Thermo Fisher Scientific, Waltham, MA, USA) with an infra-red microscope (Nicolet 6700, Thermo Fisher Scientific, Waltham, MA, USA) while m.p. was measured using a Mettler Toledo Melting Point System (MP 50) (Mettler-Toledo AG, Schwerzenbach, Swizerland).

### 3.3. Synthesis of [2′,3′,5′,6′-D_4_]Naringenin

As shown in Figure 1, 0.5 g (0.85 mmol) [2′,3′,5′,6′-D_4_]naringin (**2**) [18] was dissolved in 50 mL (2%) sulfuric acid solution and heated at 80 °C for 40 min. Then, 30% sodium hydroxide solution was added to adjust the solution to neutral. After this, ethyl acetate was added to the extract three times. The combined organic fractions were collected, dried with Na_2_SO_4_, and concentrated under reduced pressure. Finally, the resulting residue was purified by flash column chromatography on silica gel to afford [2′,3′,5′,6′-D_4_]naringenin (**1**) as a yellow powder (75 mg, 32% yield).

### 3.4. Synthesis of 3-(4′-Hydroxyphenyl)-[2′,3′,5′,6′-D_4_]Propanoic Acid

To a stirred solution of [2′,3′,5′,6′-D_4_]bromophenol (**5**) (0.5 g, 2.8 mmol), acrylic acid (0.3 g, 4.2 mmol) and bis(triphenylphosphine)palladium(II) chloride (15.2% Pd, 30 mg) in water (20 mL) was added potassium carbonate (0.6 g, 4.3 mmol) under a nitrogen atmosphere. The reaction was heated to 80 °C and stirred for 3 h. Ethyl acetate was added and extracted three times. The combined organic fractions were collected, dried with Na_2_SO_4_, and concentrated under reduced pressure. The resulting residue (crude 4-hydroxy-[2′,3′,5′,6′-D_4_]cinnamic acid) was filtered through a short pad of silica gel and used directly without further purification. Pd/C (30 mg, 30 *wt*%) was added to a stirred solution of crude 4-hydroxy-[2′,3′,5′,6′-D_4_]cinnamic acid in methanol (20 mL) and then hydrogen was introduced for stirring overnight. The mixture was filtered through a short pad of celite and concentrated under reduced pressure. The resulting residue was purified by flash column chromatography on silica gel to afford 3-(4′-hydroxyphenyl)-[2′,3′,5′,6′-D_4_]propanoic acid (290 mg, 61% yield over two steps) as a white powder.

### 3.5. Preparation of Stock Solutions, Calibration Standards, and Quality Control Samples

All the analytical stock solutions of D_4_-NG, D_4_-NE, D_4_-HPPA, and the internal standard D_5_-BA were prepared in dissolution with methanol/water (60%:40%, *v*/*v*) at concentrations of 1 mg mL^−1^ and stored at 4 °C before use. All stock solutions were diluted to 10 μg mL^−1^ to investigate the most optimal MS/MS parameters including MRM transition, dwell time, fragmentor voltage, and collision energy.

Working standard solutions were prepared by dilution from stock solutions with 60% methanol (*v*/*v*). Ten microliter working standard solutions were spiked into an Eppendorf tube and evaporated under a gentle N_2_ stream at 37 °C followed by addition of 100 μL blank gut microbiota solution with vortex for 3 min to yield calibrated standards at concentrations of 10, 20, 50, 100, 150, 200, 500, 1000, 1500, and 2000 ng mL^−1^ for D_4_-NG; 5, 10, 25, 50, 75, 100, 250, 500, 750, and 1000 ng mL^−1^ for D_4_-NE; and 2.5, 5, 12.5, 25, 37.5, 50, 125, 250, 375, and 500 ng mL^−1^ for D_4_-HPPA.

QC samples were prepared at 20, 200, and 1500 ng mL^−1^ for D_4_-NG; 10, 100, and 750 ng mL^−1^ for D_4_-NE; and 7.5, 50, and 375 ng mL^−1^ for D_4_-HPPA, which was consistent with the preparation procedures as calibrated standards.

### 3.6. Recruitment of Human Participants

Under authorization and supervision by the Ethics Committee of the School of Life Sciences, Sun Yat-sen University (Guangzhou, China), a total of 30 healthy adult participants were recruited according to our previous study [18]. The investigation enrolled 16 female and 14 male subjects, with ages ranging from 20 to 40 and body mass indexes (BMIs) between 19 to 25 kg m^−2^. None of the participants used any alcohol, medication, or antibiotic therapies, nor did they have any accurate or chronic gastrointestinal diseases during the last three months before recruitment. Experimental design and criteria were introduced to the recruited participants in detail and signatures of informed consent were obtained.

### 3.7. Feces Sample Collection and Gut Microbiota Solution Preparation

Feces samples were freshly collected and processed for no longer than 2 h. For gut microbiota solution preparation, 1.0 g fecal samples were thoroughly homogenized with 4 mL sterilized saline solution and the suspensions were centrifuged at 100× *g* at 4 °C for 10 min to filter out food residue. One milliliter supernatant was mixed with 9 mL sterile GAM broth with a gentle vortex, followed by anaerobic (2%, 20%, and 78% for H_2_, CO_2_, and N_2_, respectively) co-incubation at 37 °C for 24 h.

### 3.8. Anaerobic Incubation of D_4_-NG with Gut Microbiota Solution

Ten microliters of D_4_-NG solution at a concentration of 342 μmol L^−1^ were added to 990 μL gut microbiota solution and vortexed softly, followed by co-incubation in anaerobic an incubator (2% H_2_, 20% CO_2_, and 78% N_2_) at 37 °C for 4, 8, 12, and 24 h, respectively.

### 3.9. Sample Preparation

Ten microliters of internal standard solution (10 μg mL^−1^) were mixed with 100 μL co-incubated solution and then the mixture was extracted by 1 mL ethyl acetate with vortexing for 3 min and centrifugation at 13,000× *g* for 15 min at 4 °C. After the liquid-liquid extraction, 900 μL supernatant was evaporated under a gentle nitrogen stream at 37 °C. The residue was re-dissolved by 100 μL 60% methanol (*v/v*) followed by vortexing for 3 min and centrifugation 15,000× *g* for 10 min at 4 °C. Five microliter supernatant was injected for RRLC-MS/MS analysis.

### 3.10. RRLC-MS/MS Conditions

A connected system of 1200 RRLC-6410 triple quadrupole (QQQ) mass spectrometers (Agilent Technology, Santa Clara, CA, USA) with an ESI was employed for RRLC-MS/MS analysis. A Poroshell 120 EC-C18 column (Agilent Technology, 3.0 × 50 mm, 2.7 μm) was applied to the chromatographic separation. The mobile phase system consisted of deionized water (A) and methanol (B). Both of the solvents A and B were with 0.1% formic acid (*v*/*v*). The separation was conducted with a linear gradient elution profile using 0–3 min: 60–100% B with a 7 min post run for system equilibration. The flow rate was kept at 0.3 mL min^−1^ and the column temperature was maintained at 40 °C.

For higher sensitivity, optimized MS/MS parameters including gas temperature, gas flow, nebulizer, and capillary were set at 350 °C, 10 L min^1^, 25 psi, and 4000 V, respectively. MRM detection mode was applied to quantify the target compounds and the optimal parameters including MRM transition, dwell time, fragmentor voltage, and collision energy, which are shown in Table 1.

### 3.11. Method Validation

This quantitative analysis method was fully validated according to the guidance of the Chinese Pharmacopoeia Commission for Bioanalytical Method Validation criteria on specificity, linearity, precision and accuracy, extract recovery, and matrix effects, which included various aspects to evaluate a new method. In addition, we applied the Guide to the Expression of Uncertainty in Measurement (GUM), International Organisation for Standardisation and the method reported by Konieczka et al. [47] to further possess detailed calculations of the different uncertainties. The results will be reported in due course.

For specificity validation, chromatograms of blank gut microbiota solutions from six individual sources were compared with samples spiked with analytes an LLOQ concentration and IS standard. The responses of any interferences should be below the 20% responses of the target analytes and the 5% response of IS.

Linearity was evaluated based on three duplicate calibrated curves including LLOQ ranging from 10–2000, 5–1000, and 2.5–500 ng mL^−1^ for D_4_-NG, D_4_-NE, and D_4_-HPPA, respectively, with an accepted accuracy of 85–115% and 20–120% for LLOQ.

Precision and accuracy were evaluated using both intra-day and inter-day analysis with four calibrated standards including LLOQ and QC samples in six duplicates with concentrations at 10, 20, 200, and 1500 ng mL^−1^ for D_4_-NG; 5, 10, 100, and 750 ng mL^−1^ for D_4_-NE; and 2.5, 7.5, 50, and 375 ng mL^−1^ for D_4_-HPPA. The precision was expressed as RSDs (%) and the accuracy was determined using RE values (%) from measured concentrations to nominal concentrations. The accepted criteria of precision RSD was less than 15% for QC samples and 20% for LLOQ samples while the qualified requirement of accuracy was RE within ±15% and ±20% for QC and LLOQ samples, respectively.

Extract recovery of analytical compounds was validated by comparing the responses of LLOQ and QC samples prepared in accordance with analytical procedures and the blank gut microbiota solution extracted samples post-spiked with working standard solutions with corresponding concentrations in six duplicates.

Matrix effects were determined by comparison of the matrix factors normalized by IS from six individual gut microbiota solution extracted samples post-spiked with low and high concentrations to corresponding neat working standard solutions in three duplicates.

Stability validation was carried out through measuring low and high QC samples in three duplicates under conditions including freeze and thaw (three cycles from −80 °C to 25 °C), short-term storage (24 h at 25 °C), and long-term storage (3 months at −80 °C).

## 4. Conclusions

In summary, this research has quantitatively analyzed naringin and its major metabolites mediated by human gut microbiota with efficiency and precision by utilizing our newly developed and validated strategy, which combines stable isotope deuterium-labeling and RRLC-MS/MS method together. As expected, our strategy not only eliminated strong matrix interferences but also showed its potential application in the research of flavonoid and phenolic acid molecules. In addition, this work has revealed that bioactive naringenin and HPPA are major metabolites of naringin under the atmosphere of human gut microorganisms, indicating that microbiota play a crucial role in regulating the pharmacological effects of naringin. In addition, our results will be of value to complement and guide the study of mass balance and pharmacokinetics in clinical research.

## Figures and Tables

**Table 1:** Multiple reaction monitoring (MRM) parameters for [2′,3′,5′,6′-D_4_]naringin (D_4_-NG), [2′,3′,5′,6′-D_4_]naringenin (D_4_-NE), 3-(4′-hydroxyphenyl)-[2′,3′,5′,6′-D_4_]propanoic acid (D_4_-HPPA), and internal standard benzoic-[2,3,4,5,6-D_5_] acid (D_5_-BA).

Compound	Retention Time (Min)	MRM Transition	Dwell Time (ms)	Fragmentor Voltage (V)	Collision Energy (V)
D_4_-NG	1.6	583.2→275.1	250	215	34
D_4_-NE	2.3	275.0→151.0	250	125	12
D_4_-HPPA	1.7	169.1→125.0	250	70	7
D_5_-BA	2.2	126.0→82.1	250	75	8

**Table 2:** Precision, accuracy, and extract recovery of D_4_-NG, D_4_-NE, and D_4_-HPPA in human gut microbiota solution. Legend: RSD, relative standard deviation; RE, relative error.

Compound	Concentration (ng mL^−1^)	Intra-Day (*n* = 6)		Inter-Day (*n* = 18)		Recovery (%, *n* = 6)
		RSD (%)	RE (%)	RSD (%)	RE (%)	Mean ± SD
**D_4_-NG**	10	0.91	7.98	1.58	6.73	57.60 ± 1.34
	20	3.00	−2.92	3.25	−6.25	57.78 ± 0.80
	200	3.58	6.08	8.20	−4.04	56.22 ± 0.89
	1500	2.24	4.98	3.55	9.81	58.02 ± 1.43
D_4_-NE	5	2.51	−2.47	4.16	0.10	53.97 ± 1.56
	10	3.49	4.54	4.38	−5.15	54.69 ± 1.78
	100	1.63	2.50	6.41	−4.78	54.97 ± 0.85
	750	1.25	−1.46	5.57	−0.25	58.50 ± 1.63
D_4_-HPPA	2.5	9.90	5.51	11.39	−2.44	49.53 ± 5.23
	7.5	4.75	8.73	7.40	3.71	59.16 ± 0.65
	50	5.91	8.69	4.46	8.30	61.71 ± 0.91
	375	1.45	−9.42	2.24	−8.90	70.69 ± 0.83

**Table 3:** Matrix effects of D_4_-NG, D_4_-NE, and D_4_-HPPA in human gut microbiota solution (mean ± SD, *n* = 3).

Matrix Resource	D_4_-NG Concentration (ng mL^−1^)		D_4_-NE Concentration (ng mL^−1^)		D_4_-HPPA Concentration (ng mL^−1^)	
	20	1500	10	750	7.5	375
1	100.8 ± 3.77	103.8 ± 8.60	99.41 ± 3.24	104.0 ± 9.11	99.86 ± 1.35	98.12 ± 7.43
2	96.53 ± 4.76	101.9 ± 5.90	98.44 ± 4.99	103.7 ± 5.60	99.27 ± 6.15	104.2 ± 4.69
3	98.28 ± 1.71	103.5 ± 3.41	100.3 ± 2.30	102.4 ± 3.88	100.4 ± 2.50	104.3 ± 3.26
4	100.8 ± 1.18	106.6 ± 3.92	100.6 ± 3.83	107.1 ± 3.57	101.1 ± 1.20	100.7 ± 3.23
5	94.73 ± 8.36	101.8 ± 4.52	97.50 ± 5.19	102.9 ± 6.48	98.25 ± 5.53	105.0 ± 4.29
6	96.42 ± 4.04	104.5 ± 7.40	96.33 ± 3.42	105.8 ± 11.19	100.4 ± 2.02	103.6 ± 2.51
RSD (%)	2.66	1.74	1.70	1.70	1.09	2.66

**Table 4:** Stability analysis of D_4_-NG, D_4_-NE, and D_4_-HPPA under various conditions (mean ± SD, *n* = 3).

Compound	Concentration (ng mL^−1^)	Freeze and Thaw (3 Cycles)	Short-Term (24 h at 25 °C)	Long-Term (3 Months at −80 °C)
D_4_-NG	20	97.82 ± 1.28	103.5 ± 0.67	107.3 ± 2.51
	1500	99.36 ± 2.62	106.6 ± 1.96	96.54 ± 3.16
D_4_-NE	10	96.17 ± 1.33	99.88 ± 1.24	104.8 ± 1.86
	750	102.6 ± 0.29	96.38 ± 2.33	101.3 ± 2.33
D_4_-HPPA	7.5	106.4 ± 1.37	105.7 ± 1.52	106.5 ± 3.12
	375	97.85 ± 2.54	106.8 ± 3.01	94.84 ± 2.86
